# Machine Learning-Based Prediction of Shoulder Dystocia in Pregnancies Without Suspected Macrosomia Using Fetal Biometric Ratios

**DOI:** 10.3390/jcm14155240

**Published:** 2025-07-24

**Authors:** Can Ozan Ulusoy, Ahmet Kurt, Ayşe Gizem Yıldız, Özgür Volkan Akbulut, Gonca Karataş Baran, Yaprak Engin Üstün

**Affiliations:** 1 Department of Perinatology, Ankara Etlik City Hospital, Ankara 06170, Turkey; akbulutvolkan@yahoo.com; 2 Department of Obstetrics and Gynecology, Ankara Etlik City Hospital, Ankara 06170, Turkey; mflkurt@gmail.com; 3 Department of Obstetrics and Gynecology, Ankara Etlik Zubeyde Hanim Women’s Health Training and Research Hospital, Ankara 06070, Turkey; drgizem.yildiz3@gmail.com (A.G.Y.); goncabaran@gmail.com (G.K.B.); ustunyaprak@yahoo.com (Y.E.Ü.)

**Keywords:** shoulder dystocia, machine learning, fetal biometric ratios, predictive modeling, obstetric risk evaluation

## Abstract

**Objective**: Shoulder dystocia (ShD) is a rare but serious obstetric emergency associated with significant neonatal morbidity. This study aimed to evaluate the predictive performance of machine learning (ML) models based on fetal biometric ratios and clinical characteristics for the identification of ShD in pregnancies without clinical suspicion of macrosomia. **Methods**: We conducted a retrospective case-control study including 284 women (84 ShD cases and 200 controls) who underwent spontaneous vaginal delivery between 37 and 42 weeks of gestation. All participants had an estimated fetal weight (EFW) below the 90th percentile according to Hadlock reference curves. Univariate and multivariate logistic regression analyses were performed on maternal and neonatal parameters, and statistically significant variables (*p* < 0.05) were used to construct adjusted odds ratio (aOR) models. Supervised ML models—Logistic Regression (LR), Random Forest (RF), and Extreme Gradient Boosting (XGB)—were trained and tested to assess predictive accuracy. Performance metrics included AUC-ROC, sensitivity, specificity, accuracy, and F1-score. **Results**: The BPD/AC ratio and AC/FL ratio markedly enhanced the prediction of ShD. When added to other features in RF models, the BPD/AC ratio got an AUC of 0.884 (95% CI: 0.802–0.957), a sensitivity of 68%, and a specificity of 83%. On the other hand, the AC/FL ratio, along with other factors, led to an AUC of 0.896 (95% CI: 0.805–0.972), 68% sensitivity, and 90% specificity. **Conclusions**: In pregnancies without clinical suspicion of macrosomia, ML models integrating fetal biometric ratios with maternal and labor-related factors significantly improved the prediction of ShD. These models may support clinical decision-making in low-risk deliveries where ShD is often unexpected.

## 1. Introduction

Shoulder dystocia (ShD) is a complication of vaginal delivery in which the fetal shoulders fail to deliver spontaneously after the head emerges. While it is relatively uncommon, it presents considerable hazards. Determining its exact incidence is challenging due to variations in definitions across the literature and inconsistencies in documentation. Estimates indicate that ShD occurs in between 0.15% and 2.0% of all births [1,2].

ShD is particularly concerning due to its association with severe neonatal complications such as brachial plexus injury (1–20%), cranial nerve trauma, perinatal mortality, damage to cranial nerves that could cause cerebral palsy, and even death of the fetus [1,3]. Several antepartum risk factors have been identified, such as fetal macrosomia, gestational diabetes mellitus, maternal obesity, and excessive gestational weight gain. In addition, intrapartum risk factors, including prolonged deceleration phase, failed or arrested descent, prolonged second stage of labor, and precipitous second stage, have been linked to an increased risk of ShD [4,5,6,7,8].

Despite advancements in ultrasound technology, recognizing fetal macrosomia—a critical risk factor for shoulder dystocia (ShD)—remains challenging in the later stages of pregnancy. Estimating fetal weight, particularly in large fetuses, is prone to considerable measurement error, and ultrasonography does not consistently provide accurate weight predictions [9,10]. Consequently, relying solely on elevated estimated fetal weight to recommend cesarean delivery is neither economically sustainable nor clinically justified, as it may lead to a high rate of unnecessary surgical interventions [9,11,12]. Moreover, nearly half of ShD cases occur in fetuses weighing less than 4000 g, indicating that weight-based thresholds alone are insufficient to prevent the majority of cases [11,13,14]. Given this context, our study specifically focused on pregnancies without clinical suspicion of macrosomia, defined as an estimated fetal weight below the 90th percentile according to Hadlock reference curves. This approach aims to improve the real-world applicability of our findings by enhancing ShD prediction in a population that would not otherwise be considered at elevated risk.

The literature has explored alternative approaches for predicting ShD due to the limitations of using fetal weight alone. Burkhardt et al. attempted to predict ShD using the difference between abdominal diameter and biparietal diameter, while Gerber et al. evaluated the abdominal/head circumference ratio as a predictive marker. However, the sensitivity and positive predictive values of these models have been limited, providing only modest clinical utility. Consequently, the inability to accurately predict ShD has led to a sense of pessimism in the literature [15,16]. While ShD is often regarded as an unpredictable and therefore unavoidable obstetric emergency [17,18], a detailed evaluation of antenatal risk factors and careful monitoring during labor may enhance prediction. The predictive potential of these factors, especially in pregnancies without clinical suspicion of macrosomia, could be further improved through machine learning techniques. Our study aims to explore whether machine learning can improve the prediction of ShD by using fetal biometric measurements and known risk factors in a population not typically classified as high-risk.

## 2. Materials and Methods

This retrospective case-control study was conducted at Etlik Zübeyde Hanım Women’s Health Training and Research Hospital between January 2017 and January 2024. Ethical approval was obtained from the institutional ethics committee with protocol number 11/2024-12-06. The study was conducted in accordance with the Declaration of Helsinki.

A total of 284 participants were included in the study, consisting of 84 cases with ShD and 200 controls. We performed a power analysis ahead of time using G*Power 3.1, based on the effect size from the study by Burkhardt and others [15]. The analysis showed that at least 84 participants (42 in each group) are needed to have strong statistical results, using a confidence level of 0.05 and a power of 0.95. This confirms the adequacy of our sample size for detecting meaningful differences between the groups. Controls were selected retrospectively from women who met all inclusion and exclusion criteria, delivered during the same time period, and did not experience shoulder dystocia. A 1:2 matching ratio was applied, with two consecutive eligible controls selected for each case to minimize selection bias and ensure temporal comparability.

The inclusion criteria were as follows: women aged 18 to 45 years, carrying a singleton fetus in vertex presentation, and scheduled for vaginal delivery. Eligible participants were retrospectively identified from medical records based on clinical data and ultrasound assessments performed during obstetric consultations between 37 and 42 weeks of gestation. Only those who were initially planned for spontaneous vaginal delivery and subsequently delivered vaginally without cesarean section were included in the final analysis.

The exclusion criteria included cesarean delivery, preterm birth (gestational age < 37 weeks), breech presentation, suspected macrosomia (defined as an estimated fetal weight above the 90th percentile for gestational age), and intrauterine fetal demise.

The maternal and neonatal data were obtained from the hospital’s electronic medical record system. Fetal measurements, including biparietal diameter (BPD), abdominal circumference (AC), and femur length (FL), were recorded, and estimated fetal weight (EFW) was calculated using the Hadlock formula [19]. All ultrasound measurements were performed between 37 and 42 weeks of gestation during the admission visit for planned vaginal delivery. In line with the study’s inclusion criteria, patients with an EFW above the 90th percentile for gestational age, according to Hadlock growth standards, were excluded due to clinical suspicion of macrosomia. Ultrasound measurements were performed using the Voluson 730 (GE Healthcare, Chicago, IL, USA) ultrasound system.

ShD was defined as the requirement for additional obstetric maneuvers beyond gentle downward traction after delivery of the fetal head to achieve complete birth. In all cases, the initial approach was the McRoberts maneuver, followed by the Jacquemier maneuver, if necessary [20,21,22]. When these failed, we applied rotational maneuvers like the Woods screw, Rubin, or Gaskin to facilitate delivery [23].

The duration of labor was recorded in stages. Stage 1 duration referred to the active phase of labor, measured from 4–6 cm cervical dilatation to full dilatation at 10 cm. Stage 2 duration was defined as the time interval from complete cervical dilatation (10 cm) to the delivery of the fetus.

The Composite Adverse Perinatal Outcome (CAPO) was used as a secondary outcome measure to assess neonatal morbidity. CAPO was defined as the presence of at least one of the following: an APGAR score below 7 at both 1 and 5 min, neonatal intensive care unit (NICU) admission, or documented brachial plexus injury.

## 3. Statistical Analysis

Normality of continuous variables was assessed using the Kolmogorov–Smirnov test. Continuous variables following a normal distribution were analyzed using Student’s *t*-test, while those not normally distributed were compared using the Mann–Whitney U test. Categorical variables were analyzed using the chi-square test. Continuous variables are presented as median values with interquartile ranges (25th and 75th percentiles), while categorical variables are presented as frequencies and percentages (n, %). Univariate logistic regression was performed for all independent variables to assess their association with ShD, and those with *p* < 0.05 were included in a multivariate logistic regression model to obtain adjusted odds ratios (aOR) and 95% confidence intervals (CIs). Additionally, estimated marginal means (EMMs) were derived from the logistic regression model to adjust for covariates and further examine group differences.

**Analysis of Machine Learning**

Supervised machine learning approaches were employed to improve predictive modeling, with the dataset divided into training (70%) and test (30%) sets. The employed models were as follows: 1. Logistic Regression (LR), 2. Random Forest Classifier (RF), 3. Extreme Gradient Boosting (XGB).

***Feature Selection Strategy:*** The selection of features for the machine learning models was predicated on factors deemed significant in univariate logistic regression (*p* < 0.01) and fetal biometric ratios exhibiting the highest sensitivity for predicting ShD. Hyperparameter optimization was performed using GridSearchCV with 5-fold cross-validation to enhance model performance, while Synthetic Minority Over-sampling Technique (SMOTE) was employed to address class imbalance, creating a more balanced and representative training dataset.

***Assessment of Performance:*** The model’s performance was assessed on the test set utilizing various metrics, such as area under the receiver operating characteristic curve (AUC-ROC), sensitivity, specificity, accuracy, and F1-score. Furthermore, bootstrapping with 1000 resamples was conducted to calculate 95% confidence intervals for AUC, thus assuring rigorous statistical validation of the model’s discriminative capacity.

**Figure 1:**
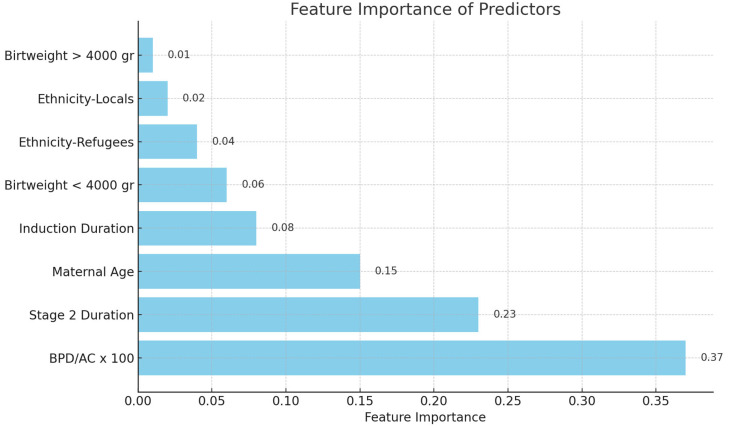
Feature importance in shoulder dystocia prediction using the BPD/AC ratio.

**Figure 2:**
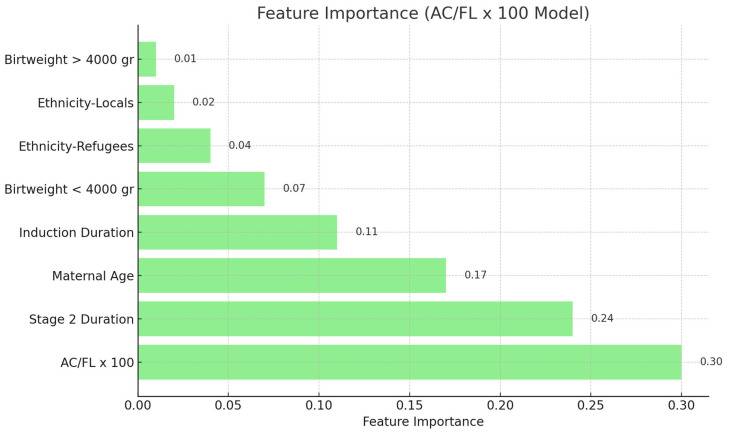
Feature importance in shoulder dystocia prediction using the AC/FL ratio.

***Analysis of Feature Importance:*** The feature significance values of the Random Forest model were extracted and ranked to identify the most significant predictors (Figure 1 and Figure 2). All statistical tests were conducted as two-tailed, with *p* < 0.05 being statistically significant.

## 4. Results

Of the 284 participants included, 84 experienced shoulder dystocia (ShD) and 200 served as controls. Maternal age was significantly higher in the ShD group (median 27.5 vs. 26.0 years, *p* = 0.010), while parity and nulliparity rates showed no significant difference. Ethnic distribution differed between groups (*p* = 0.021), with a higher proportion of refugee patients in the control group. Gestational diabetes was significantly more prevalent among those with ShD (16.6% vs. 0.5%, *p* < 0.001). Ultrasound parameters showed significantly larger fetal biometric values in the ShD group, including BPD (94.0 vs. 92.0 mm), AC (348.0 vs. 331.0 mm), FL (74.0 vs. 72.0 mm), and EFW (3600 vs. 3210 g) (all *p* < 0.001). Labor characteristics revealed a lower rate of labor induction in the ShD group (73.8% vs. 90.5%, *p* < 0.001), but higher episiotomy rates (80.9% vs. 46.5%, *p* < 0.001) and prolonged durations of the second and third stages of labor (both *p* < 0.001). Birthweight was significantly higher in the ShD group (3873 vs. 3180 g, *p* < 0.001), with 36.9% of neonates weighing over 4000 g, compared to 3.5% in the control group. While gestational age at birth was comparable between the groups (*p* = 0.166), the incidence of composite adverse perinatal outcomes—including low Apgar scores, NICU admission, and brachial plexus injury—was markedly higher in the ShD group (57.1% vs. 0%, *p* < 0.001) (Table 1).

Supplement Table S1 indicates that McRoberts and suprapubic pressure were routinely performed (100%) in cases of ShD, but additional techniques such as Jacquemier (27.4%), Rubin (14.3%), and Woods (14.3%) were utilized selectively. The median number of maneuvers performed was 2.0 (IQR: 2.0–3.0). Brachial plexus injury occurred in 45.2% of instances, highlighting the considerable newborn morbidity linked to ShD.

Table 2 shows the outcomes of univariate and multivariate logistic regression studies for factors linked to ShD. In univariate logistic regression analysis, maternal age, ethnicity (refugees vs. locals), birth weight > 4000 g, stage 2 duration at birth, and labor induction duration were significantly associated with shoulder dystocia (*p* < 0.05). In multivariate analysis, birth weight > 4000 g (aOR: 51.668, 95% CI: 16.690–159.95, *p* < 0.001), stage 2 duration at birth (aOR: 1.035, 95% CI: 1.010–1.060, *p* = 0.006), and labor induction duration (aOR: 1.003, 95% CI: 1.000–1.010, *p* = 0.027) remained independently significant predictors.

Table 3 shows the diagnostic efficacy of fetal biometric ratios in predicting ShD. The BPD/AC ratio100 exhibited the greatest AUC (0.855) of the analyzed variables, with a sensitivity of 65% and specificity of 90%. The EFW/BPD ratio and EFW/FL ratio had strong predictive capabilities, exhibiting AUC values of 0.828 and 0.810, respectively. Although EFW > 4000 g did not achieve statistical significance (*p* = 0.067), the EFW/AC ratio (AUC: 0.770) and AC/FL ratio100 (AUC: 0.757) were significant predictors. These findings highlight the significance of fetal biometric ratios compared to absolute weight in evaluating the probability of ShD.

Table 4 shows the efficacy of machine learning models in predicting ShD by utilizing biometric ratios alongside additional clinical characteristics. The AC/FL ratio combined with additional features using Random Forest attained the best specificity of 90% and an AUC of 0.896 (95% CI: 0.805–0.972). The BPD/AC ratio and other features utilizing Random Forest exhibited robust predictive efficacy, with an AUC of 0.884 (95% CI: 0.802–0.957) with 68% sensitivity and 83% specificity. The integration of biometric ratios into the models enhanced their overall performance, especially sensitivity, in contrast to models that utilized solely clinical features.

Table 5 shows the predicted likelihood of ShD according to the BPD/AC ratio and AC/FL ratio. A diminished BPD/AC ratio correlates with a heightened likelihood of ShD exhibiting a chance of 61.4% (95% CI: 51.2–70.6%) at 26.8, which declines to 4.5% (95% CI: 2.3–8.4%) at 28.2. A greater AC/FL ratio correlates with an elevated risk, with probabilities escalating from 11.1% (95% CI: 6.9–17.2%) at 456 to 49.9% (95% CI: 40.8–59.0%) at 476. These findings highlight the potential of biometric ratios to improve ShD risk evaluation.

Feature importance analysis revealed that the BPD/AC ratio possessed the greatest predictive value for ShD among the assessed biometric ratios (Figure 1). In the Random Forest model utilizing this ratio, the BPD/AC ratio accounted for 37% of the total prediction. In the model using the AC/FL ratio, this variable had a high significance at 30% (Figure 2). Moreover, Stage 2 duration and mother age were consistently significant factors in both models. Feature importance values were normalized such that the sum of all features’ importances approximated 1.0. Due to rounding, the reported percentages may not total exactly 100%. These values reflect each feature’s relative contribution to the model’s predictive capacity.

## 5. Discussion

Our study showed that using the BPD/AC ratio and AC/FL ratio along with other features can help effectively predict shoulder dystocia (ShD) when using machine learning (ML) methods. The BPD/AC ratio had a sensitivity of 68%, a specificity of 83%, and an AUC of 0.884 (with a 95% confidence interval of 0.802 to 0.957). The AC/FL ratio also had a sensitivity of 68%, a specificity of 90%, and an AUC of 0.896 (with a 95% confidence interval of 0.805 to 0.972).

Importantly, our analysis specifically focused on pregnancies without clinical suspicion of macrosomia, defined as estimated fetal weight (EFW) below the 90th percentile for gestational age according to Hadlock reference curves. This design decision was deliberate in order to enhance clinical relevance by addressing ShD risk in a population typically regarded as low-risk and not eligible for preemptive intervention such as elective cesarean delivery.

Previous studies have attempted to predict ShD using various fetal biometric measurements. Gerber et al. reported that an AC/HC ratio of ≥1.05 had a sensitivity of 46.2% and a specificity of 74.6% for predicting ShD [16]. Burkhardt et al. found that the difference between AD and BPD (AD–BPD) (AUC = 0.704) significantly increased the risk of ShD (OR: 7.6, 95% CI: 4.2–13.9), with a sensitivity of only 8.2% and a specificity of 98.8%. The positive predictive value (PPV) was low at 7.5%, which led the authors to decide that using only ultrasound measurements of the fetus is not enough for making accurate diagnoses [15]. In contrast, our ML-based models improved the performance of both the BPD/AC and AC/FL ratios in predicting ShD, achieving sensitivities between 53 and 63% and specificities between 76 and 91%. These results align with other ML-based predictive models.

Another relevant study by Futterman et al. used an ML model to predict neuroskeletal injury secondary to ShD. Their model incorporated head-to-body time, maternal BMI, neonatal birthweight, oxytocin use, and type of delivery as independent variables, achieving a sensitivity of 73.4%, specificity of 70.3%, an AUC of 0.76 (95% CI: 0.70–0.81, Figure 1), and an F1-score of 0.28 [24]. However, their model did not include fetal biometric measurements. While our study yielded similar sensitivity results, our specificity and F1 scores were higher.

One of the major limitations in predicting ShD is the inaccuracy of fetal weight estimation (EFW) in advanced gestational weeks, despite improvements in ultrasound technology [25]. Tsur et al. demonstrated that an ML model using EFW alone effectively classified ShD risk and related neonatal injuries in women carrying fetuses ≥ 4000 g [26]. However, EFW alone may fail to predict ShD in macrosomic fetuses that are not sonographically suspected of macrosomia. This discrepancy is particularly important considering that the observed–predicted EFW difference can have a standard error of up to 15%. While improving the accuracy of EFW estimations could help, it may also lead to an increased rate of unnecessary cesarean deliveries.

Another potential limitation of our study is that fetal biometric ratios were not adjusted for gestational age. Although all measurements were obtained between 37 and 42 weeks, these ratios may still vary within this interval due to subtle fetal growth changes. Future research should consider gestational age-standardized Z-scores or percentile-based thresholds to enhance model precision.

Additionally, to address the class imbalance between ShD cases and controls, we applied the Synthetic Minority Over-sampling Technique (SMOTE) to the training data. While this improved sensitivity, it also introduces potential limitations, including the risk of overfitting and reduced generalizability in external datasets. Thus, external validation in a larger, independent cohort remains a necessary step for future model optimization.

Machine learning analyses inherently necessitate substantial sample sizes for optimal efficacy. A weakness of our work is the relatively small sample size and inadequate representation of patients with gestational diabetes in the control group, resulting in their exclusion from machine learning studies. Furthermore, a significant factor contributing to ShD—the acceleration and deceleration phases of the initial stage of labor—was not examined individually, which constitutes an additional drawback of the study. Future research utilizing larger sample sizes, maternal pelvimetry assessments, and a comprehensive investigation of the initial stage of labor will likely improve the predictive precision of machine learning models for ShD.

Despite these limitations, our research represents one of the early attempts to employ machine learning techniques in predicting ShD, a condition associated with significant neonatal problems and a prominent concern for healthcare professionals. One of the strengths of our study is the inclusion of only non-macrosomic fetuses based on sonographic estimates, which enhances its clinical applicability. Moreover, by combining labor progression data with sonographic indicators, our methodology offers a more exhaustive predictive model than prior research.

## 6. Conclusions

Our study highlights the potential of machine learning (ML) models in enhancing the prediction of ShD (ShD) by integrating fetal biometric ratios with clinical features. The BPD/AC and AC/FL ratios, when combined with other variables, significantly improved predictive performance, surpassing traditional sonographic assessments. 

## Figures and Tables

**Table 1:** Comparison of demographic, ultrasonographic, and neonatal characteristics between the shoulder dystocia and control groups.

	Shoulder Dystocia (n:84)	Control (n:200)	*p* Value
**Demographics**, median (IQR)—n (%)			
**Maternal age in years**	27.5 (24.0, 32.0)	26.0 (23.0, 30.0)	**0.010**
**Parity**	1.0 (0.0, 2.0)	1.0 (0.0, 2.0)	0.147
**Nulliparity**	24 (28.5)	51 (25.5)	0.576
**Maternal ethnicity**			**0.021**
Locals	71 (84.5)	144 (72.0)	
Refugees	13 (15.5)	56 (28.0)	
**Maternal body mass index in Kg/m^2^**	30.0 (27.0, 34.0)	30.0 (27.0, 33.0)	0.343
**Gestational diabetes**	14 (16.6)	1 (0.5)	**<0.001**
**Ultrasonographic findings at scan**, median (IQR)			
**BPD at ultrasound (mm)**	94.0 (92.0, 95.0)	92.0 (91.0, 94.0)	**<0.001**
**AC at ultrasound (mm)**	348.0 (344.0, 352.0)	331.0 (327.0, 338.0)	**<0.001**
**FL at ultrasound (mm)**	74.0 (72.0, 75.0)	72.0 (70.0, 73.0)	**<0.001**
**EFW at ultrasound (mm)**	3600 (3500, 3806)	3210 (3000, 3440)	**<0.001**
**Labor Findings**, median (IQR)—n (%)			
**Induced labor**	62 (73.8)	181 (90.5)	**<0.001**
**Stage 1 duration at birth (min)**	180 (120, 360)	180 (120, 300)	0.078
**Stage 2 duration at birth (min)**	15.0 (10.0, 30.0)	5.0 (5.0, 20.0)	**<0.001**
**Stage 3 duration at birth (min)**	5.0 (5.0, 10.0)	5.0 (5.0, 5.0)	**<0.001**
**Operative Vaginal Delivery**	1 (1.1)	2 (1.0)	0.883
**Episiotomy**	68 (80.9)	93 (46.5)	**<0.001**
**Postnatal findings**, median (IQR)—n (%)			
**Gestational age at birth in weeks**	39.1 (38.5, 39.7)	39.1 (38.2, 40.1)	0.166
**Birthweight in grams**	3873 (3600, 4070)	3180 (2990, 3435)	**<0.001**
**Birthweight > 4000 gr**	31 (36.9)	7 (3.5)	**<0.001**
**Composite Adverse Perinatal Outcome**	48 (57.1)	0 (0.0)	**<0.001**

*p* < 0.05 was statistically significant. Note: Composite Adverse Perinatal Outcome consists of an APGAR score below 7 at 1 and 5 min, NICU admission, and brachial plexus injury. BPD: bi-parietal diameter; AC: abdominal circumference; FL: femur length; EFW: estimated fetal weight; IQR: interquartile range.

**Table 2:** Factors associated with shoulder dystocia: univariate and multivariate logistic regression analysis.

	Univariate LR		Multivariate LR	
	OR (95% CI)	*p* Value	aOR (95% CI)	*p* Value
**Maternal age in years**	1.066 (1.017–1.117)	**0.008**	1.047 (0.969–1.130)	0.246
**Maternal BMI at booking**	1.037 (0.975–1.103)	0.240		
**Nulliparity**	1.176 (0.665–2.081)	0.576		
**Ethnicity (Refugees vs. Locals)**	0.463 (0.238–0.900)	**0.023**	0.440 (0.137–1.410)	0.168
**Birth Weight > 4000 gr**	16.210 (6.760–38.87)	**<0.001**	51.668 (16.690–159.95)	**<0.001**
**Stage 1 duration at birth**	1.001 (1.000–1.003)	0.108		
**Stage 2 duration at birth**	1.032 (1.015–1.048)	**<0.001**	1.035 (1.010–1.060)	**0.006**
**Labor induction duration**	1.003 (1.001–1.003)	**0.003**	1.003 (1.000–1.010)	**0.027**

*p* < 0.05 was statistically significant. OR: odds ratio; CI: confidence interval; aOR: adjusted odds ratio.

**Table 3:** Diagnostic performance of fetal biometric ratios for predicting shoulder dystocia.

	OR (95% CI)	*p* Value	R^2^	AUC	Sensitivity	Specificity
**EFW/BPD Ratio**	1.620 (1.430–1.840)	**<0.001**	0.369	0.828	46%	89%
**EFW/AC Ratio**	4.640 (2.890–7.460)	**<0.001**	0.240	0.770	34%	90%
**EFW/FL Ratio**	1.460 (1.320–1.630)	**<0.001**	0.333	0.810	39%	89%
**BPD/AC Ratio × 100**	0.080 (0.043–0.149)	**<0.001**	0.447	0.855	65%	90%
**BPD/FL Ratio × 100**	0.820 (0.748–0.900)	**<0.001**	0.094	0.670	13%	95%
**AC/FL Ratio × 100**	1.110 (1.070–1.140)	**<0.001**	0.221	0.757	46%	95%

Note: The cut-off values set to 0.5 and *p* < 0.05 is statistically significant. BPD: bi-parietal diameter; AC: abdominal circumference; FL: femur length; EFW: estimated fetal weight.

**Table 4:** Performance of ML models in predicting shoulder dystocia using biometric ratios.

Variable	Model	Sensitivity	Specificity	F1 Score	Accuracy	AUC 95% CI	Shrinkage
**Only Other Features**	*RF*	47%	88%	52%	78%	0.795 (0.678–0.896)	0.204
	*LR*	57%	77%	52%	72%	0.739 (0.594–0.853)	0.160
	*XGB*	36%	83%	40%	71%	0.718 (0.588–0.840)	0.281
**BPD/AC Ratio**	*RF*	63%	91%	70%	81%	0.802 (0.685–0.905)	0.178
	*LR*	63%	91%	70%	81%	0.827 (0.713–0.924)	0.056
	*XGB*	60%	91%	67%	80%	0.828 (0.720–0.917)	0.088
**AC/FL Ratio**	*RF*	56%	92%	66%	80%	0.819 (0.714–0.907)	0.172
	*LR*	56%	76%	56%	69%	0.724 (0.585–0.852)	0.011
	*XGB*	53%	92%	64%	79%	0.826 (0.721–0.921)	0.139
**BPD/AC Ratio** **+** **Other Features**	*RF*	68%	83%	63%	79%	0.884 (0.802–0.957)	0.113
	*LR*	68%	81%	61%	78%	0.839 (0.726–0.938)	0.112
	*XGB*	57%	87%	59%	79%	0.878 (0.786–0.954)	0.121
**AC/FL Ratio** **+** **Other Features**	*RF*	68%	90%	70%	84%	0.896 (0.805–0.972)	0.103
	*LR*	73%	79%	63%	78%	0.843 (0.719–0.942)	0.082
	*XGB*	52%	87%	55%	78%	0.807 (0.692–0.911)	0.192
**BPD/AC Ratio** **+** **Stage 2 Duration**	*RF*	63%	94%	73%	83%	0.838 (0.734–0.928)	0.155
	*LR*	66%	85%	68%	79%	0.832 (0.726–0.926)	0.060
	*XGB*	60%	91%	67%	80%	0.838 (0.724–0.934)	0.133
**AC/FL Ratio** **+** **Stage 2 Duration**	*RF*	63%	78%	62%	73%	0.810 (0.692–0.895)	0.189
	*LR*	63%	78%	62%	73%	0.730 (0.592–0.853)	0.035
	*XGB*	56%	89%	64%	77%	0.803 (0.680–0.905)	0.194

**Note:** The variables Maternal Age, Stage 2 duration, Induction duration, and Birth weight were used in machine learning models as ‘Other Features’. All performance metrics are calculated based on ‘test sets’. RF: Random Forest; LR: Logistic Regression; XGB: Extreme Gradient Boosting; AUC: area under curve; CI: confidence interval.

**Table 5:** Predicted probability of shoulder dystocia based on the BPD/AC ratio and AC/FL ratio.

				95% CI	
		Probability	SE	Lower	Upper
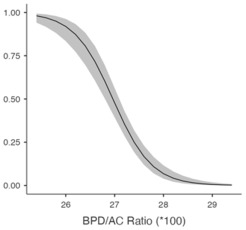	**BPD/AC Ratio (×100)**				
	26.8 ^−^	0.6135	0.0503	0.5116	0.7064
	27.5 ^μ^	0.2139	0.0308	0.1598	0.2803
	28.2 ^+^	0.0446	0.0148	0.0231	0.0843
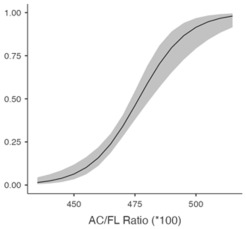	**AC/FL Ratio (×100)**				
	456 ^−^	0.111	0.0257	0.0699	0.172
	466 ^μ^	0.261	0.0289	0.2082	0.321
	476 ^+^	0.499	0.0470	0.4078	0.590

Note. ^−^ mean − 1SD, ^μ^ mean, ^+^ mean + 1SD.

## Data Availability

Due to hospital policies, patient data and study materials cannot be shared. However, the data are available from the corresponding author upon reasonable request.

## Supplementary Materials

The following supporting information can be downloaded at: https://www.mdpi.com/article/10.3390/jcm14155240/s1, Table S1. Management and Outcomes of Shoulder Dystocia.

## Abbreviations

**AC**—Abdominal Circumference, **AOR**—Adjusted Odds Ratio, **AUC**—Area Under Curve, **ROC**—Receiver Operating Characteristic, **BPD**—Biparietal Diameter, **CAPO**—Composite Adverse Perinatal Outcome, **CI**—Confidence Interval, **EFW**—Estimated Fetal Weight, **FL**—Femur Length, **LR**—Logistic Regression, **ML**—Machine Learning, **NICU**—Neonatal Intensive Care Unit, **PPV**—Positive Predictive Value, **RF**—Random Forest, **RDS**—Respiratory Distress Syndrome, **SD**—Standard Deviation, **ShD**—Shoulder Dystocia, **XGB**—Extreme Gradient Boosting.
