# Supplementary material for: Machine Learning-Based Prediction of Shoulder Dystocia in Pregnancies Without Suspected Macrosomia Using Fetal Biometric Ratios
Source: J Clin Med. 2025 Jul 24;14(15):5240. doi: 10.3390/jcm14155240 (PMC12347650; doi:10.3390/jcm14155240)
Supplement: Supplementary file 1 [file jcm-14-05240-s001.zip › jcm-3730977-supplementary.pdf]

Supplement Table S1. Management and Outcomes of Shoulder Dystocia

	**Shoulder dystocia (n:84)**
**Shoulder dystocia duration (sec),** median (IQR)	30.0 (15.0, 32.0)
**Maneuvers,** n (%)	
**McRoberts**	84/84 (100)
**Rubin**	12/84 (14.3)
**Woods**	12/84 (14.3)
**Jacquemier**	23/84 (27.4)
**Gaskin**	2/84 (2.4)
**Number of Maneuvers,** median (IQR)	2.0 (2.0, 3.0)
**Brachial plexus injury,** n (%)	38/84 (45.2)
